# Asymptomatic Cerebrospinal Fluid HIV-1 Escape: Incidence and Consequences

**DOI:** 10.1093/infdis/jiae555

**Published:** 2024-11-12

**Authors:** Gustaf Ulfhammer, Aylin Yilmaz, Åsa Mellgren, Erika Tyrberg, Erik Sörstedt, Lars Hagberg, Johanna Gostner, Dietmar Fuchs, Henrik Zetterberg, Staffan Nilsson, Kristina Nyström, Arvid Edén, Magnus Gisslén

**Affiliations:** Department of Infectious Diseases, Institute of Biomedicine, Sahlgrenska Academy, University of Gothenburg, Gothenburg, Sweden; Department of Infectious Diseases, Sahlgrenska University Hospital, Gothenburg, Sweden; Department of Infectious Diseases, Institute of Biomedicine, Sahlgrenska Academy, University of Gothenburg, Gothenburg, Sweden; Department of Infectious Diseases, Sahlgrenska University Hospital, Gothenburg, Sweden; Department of Infectious Diseases, Institute of Biomedicine, Sahlgrenska Academy, University of Gothenburg, Gothenburg, Sweden; Department of Infectious Diseases, Sahlgrenska University Hospital, Gothenburg, Sweden; Department of Infectious Diseases, Institute of Biomedicine, Sahlgrenska Academy, University of Gothenburg, Gothenburg, Sweden; Department of Infectious Diseases, Sahlgrenska University Hospital, Gothenburg, Sweden; Department of Infectious Diseases, Institute of Biomedicine, Sahlgrenska Academy, University of Gothenburg, Gothenburg, Sweden; Department of Infectious Diseases, Sahlgrenska University Hospital, Gothenburg, Sweden; Department of Infectious Diseases, Institute of Biomedicine, Sahlgrenska Academy, University of Gothenburg, Gothenburg, Sweden; Department of Infectious Diseases, Sahlgrenska University Hospital, Gothenburg, Sweden; Institute of Medical Biochemistry, Biocenter, Innsbruck Medical University, Innsbruck, Austria; Institute of Biological Chemistry, Biocenter, Innsbruck Medical University, Innsbruck, Austria; Department of Psychiatry and Neurochemistry, Institute of Neuroscience and Physiology, Sahlgrenska Academy, University of Gothenburg, Gothenburg, Sweden; Clinical Neurochemistry Laboratory, Sahlgrenska University Hospital, Mölndal, Sweden; Department of Neurodegenerative Disease, Institute of Neurology, University College London, London, United Kingdom; UK Dementia Research Institute, University College London, London, United Kingdom; Hong Kong Center for Neurodegenerative Diseases, Hong Kong, China; Wisconsin Alzheimer's Disease Research Center, School of Medicine and Public Health, University of Wisconsin-Madison, Madison, Wisconsin, USA; Department of Laboratory Medicine, Institute of Biomedicine, Sahlgrenska Academy, University of Gothenburg, Gothenburg, Sweden; Department of Infectious Diseases, Institute of Biomedicine, Sahlgrenska Academy, University of Gothenburg, Gothenburg, Sweden; Department of Infectious Diseases, Institute of Biomedicine, Sahlgrenska Academy, University of Gothenburg, Gothenburg, Sweden; Department of Infectious Diseases, Sahlgrenska University Hospital, Gothenburg, Sweden; Department of Infectious Diseases, Institute of Biomedicine, Sahlgrenska Academy, University of Gothenburg, Gothenburg, Sweden; Department of Infectious Diseases, Sahlgrenska University Hospital, Gothenburg, Sweden; Public Health Agency of Sweden, Solna, Sweden

**Keywords:** HIV-1, neopterin, immune activation, albumin ratio, neurofilament protein light

## Abstract

**Background:**

The incidence and clinical relevance of asymptomatic cerebrospinal fluid escape (CSFE) during antiretroviral therapy (ART) is uncertain. We examined the impact and incidence of asymptomatic CSFE in a Swedish HIV cohort.

**Methods:**

Neuroasymptomatic people with HIV (PWH) who have been on ART for at least 6 months with suppressed plasma viral load were followed longitudinally. CSFE was defined as either increased CSF HIV-1 RNA with concurrent plasma suppression or CSF HIV-1 RNA exceeding that in plasma when both were quantifiable. Paired CSF and plasma were analyzed for HIV-1 RNA, neopterin, neurofilament light protein (NfL), white blood cell (WBC) count, and albumin ratio.

**Results:**

Asymptomatic CSFE (cutoff 50 copies/mL) was found in 4 of 173 PWH (2%) and 5 of 449 samples (1%). The corresponding proportions were 8% of PWH and 4% for samples using a 20 copies/mL cutoff for CSF HIV-1 RNA. CSFE samples (cutoff 20 copies/mL) had a 25% higher geometric mean of CSF neopterin (*P* = .01) and 8% higher albumin ratio (*P* = .04) compared to samples without CSFE. No differences were observed in CSF NfL levels (*P* = .8). The odds ratio for increased CSF WBC (≥ 3 cells/μL) in samples with CSFE was 3.9 (*P* = .004), compared to samples without elevated CSF viral load.

**Conclusions:**

Asymptomatic CSFE was identified in only 4 (2%) PWH, with no cases of continuous CSFE observed. Increased CSF HIV-1 RNA was associated with biomarkers of CNS immune activation and blood-brain barrier impairment, but not with biomarkers of neuronal injury.

## Teaser

We examined asymptomatic CSF escape in a Swedish HIV cohort, finding it present in 2% of people with HIV. Quantifiable CSF HIV-1 RNA was associated with CNS inflammation but not neuronal injury, indicating that asymptomatic CSF escape is clinically benign.

Human immunodeficiency virus type 1 (HIV-1) invades the central nervous system (CNS) early during primary infection and can persistently be found in cerebrospinal fluid (CSF) throughout the untreated course of the infection [1–4]. Antiretroviral treatment (ART) is generally highly effective in suppressing CSF HIV-1 RNA below quantification limits of clinical assays, thereby preventing severe neurological complications such as HIV-associated dementia [5–8]. However, cases of isolated elevations of CSF HIV-1 RNA occur despite ART-mediated viral suppression in plasma, constituting a phenomenon termed CSF HIV-1 escape (CSFE) [9–17]. In recent years, CSFE has generated substantial interest due to its potential to provide valuable insights into the CNS reservoir establishment and persistence, which in turn, has implications for HIV cure approaches [18–20].

Three subgroups of CSFE have been described [9]: neurosymptomatic, secondary, and asymptomatic. Neurosymptomatic CSFE is a rare, but clinically important condition that resembles the pathological entity CD8^+^ encephalitis [9, 10, 21, 22] and is often resolved by modifications of the ART regimen, stressing the role of HIV as the primary etiological factor [10, 23]. Secondary CSFE is insufficiently studied but is most likely a result of the host's local responses to non-HIV pathogens that usually resolve without ART adjustments [15, 24].

Asymptomatic CSFE was first described by our research team where we have reported a cross-sectional incidence of CSFE of 10% [17] and a longitudinal incidence of 36% [12] in people with HIV (PWH) on suppressive ART monitored in a longitudinal research protocol. Asymptomatic CSFE was associated with elevated CSF neopterin but not CSF neurofilament light protein (NfL), was mostly sporadic, and did not lead to treatment failure. Overall, these findings indicate that most cases of asymptomatic CSFE are clinically benign, usually transient, and coupled to CNS inflammation that does not seem to cause neuronal damage [12, 17]. The incidence of CSFE varies widely across studies, ranging from 0.7% to 36%, influenced by multiple factors including the presence or absence of neurological symptoms [11–13, 17, 18, 22, 25–27], diversities in the cohorts studied, variations in polymerase chain reaction (PCR) assays, and the definitions of CSFE employed across different studies [13].

The objective of this study was to investigate the incidence of asymptomatic CSFE within a large and well-characterized Swedish cohort of PWH receiving contemporary ART. Furthermore, we aimed to identify potential predictors of asymptomatic CSFE and explore its impact on CSF biomarkers associated with CNS inflammation and injury.

## METHODS

### Study Design and Participants

The Gothenburg HIV CSF Study Cohort at Sahlgrenska University Hospital [28] includes untreated and treated PWH, both neurologically symptomatic and asymptomatic (hereafter referred to as neuroasymptomatic). For this specific study, we longitudinally followed participants from 2016 to 2022 who (1) had been on ART for more than 6 months, (2) had a plasma HIV-1 RNA level < 50 copies/mL at inclusion, (3) were neuroasymptomatic at the time of enrolment, and (4) who had undergone lumbar puncture at least once as part of the research protocol. Neuroasymptomatic state was characterized as either being entirely free of neurological symptoms or signs, or having neurological symptoms that could be attributed to stable sequelae caused by cerebrovascular events or CNS opportunistic infection > 12 months prior to inclusion. Viral plasma blips, defined as transient HIV-1 RNA between 20 and 500 copies/mL, were allowed. Treatment interruptions, defined as absence of ART > 2 weeks, or ongoing or recent CNS disease such as cerebrovascular disease, CNS neoplasms, or CNS infections within the last year were not allowed. The start of the study was based on the implementation of the COBAS 6800 (Hoffman La-Roche) as the standard assay used for routine quantification of HIV-1 RNA.

CSFE was defined according to the current European AIDS Clinical Society guidelines [29] as either CSF HIV-1 RNA ≥50 copies/mL concurrently with plasma suppression or as CSF HIV-1 RNA exceeding that of plasma if both were quantifiable, with an upper limit of plasma HIV-1 RNA of 500 copies/mL [29]. Because the lower limit of quantification (LLQ) of contemporary real-time PCR assays is 20 copies/mL, we also present the incidence when HIV-1 RNA ≥20 copies/mL was used as the cutoff. Each study visit included a clinical neurological examination. Participant characteristics were collected from medical records.

The study was approved by the Regional Ethics Review Board at the Sahlgrenska Academy, University of Gothenburg (Gothenburg, Sweden; No. Ö 588-01) and was performed in accordance with the Declaration of Helsinki. All blood and CSF samples were obtained after written informed consent.

### CSF and Blood Measurements

HIV-1 RNA levels in plasma and CSF were quantified using the COBAS 6800 system (Hoffman La-Roche), with a LLQ of 20 copies/mL. Blood CD4^+^ T-cell count, CSF white blood cell (WBC), and CSF and blood albumin measurements were conducted in the local clinical laboratory using standard clinical chemistry assays. The limit of detection for CSF WBCs was ≥ 3 × 10^6^ cells/L. The albumin ratio was calculated as CSF albumin (mg/L)/plasma albumin (g/L). Reference values were < 6.8 for individuals younger than 45 years and < 10.2 for individuals 45 years and older. Neopterin concentrations in plasma and CSF were measured using a commercially available immunoassay (NEOPT-SCR.EIA 384 Det., Thermo Fisher Scientific-BRAHMS GmbH) with an upper normal reference value of 9.1 nmol/L in plasma and 5.8 nmol/L in CSF [30, 31]. CSF NfL concentration was measured utilizing the commercial NF-light ELISA kit (UmanDiagnostics AV). All NfL values were age-adjusted to the age of 50 years. The normal reference value was set as < 990 ng/L, determined by the antilog of the log scale mean + 2 standard deviations in a cohort of 359 healthy controls [32].

### Statistical Methods

Descriptive continuous data are presented as median and interquartile range (IQR) while categorical variables are listed as numbers and percentages unless otherwise stated. For repeated measurements of CSF neopterin, albumin ratio, and CSF NfL, the median of the mean values for measurements taken from the same participant is presented (referred to as the median averaged value). Continuous variables were log_10_ transformed where appropriate for the tests used. Two-group comparisons were analyzed by Mann-Whitney *U* test for continuous variables and by Fisher exact test for dichotomous variables. Analyses involving repeated measurements of CSF NfL level, CSF and plasma neopterin levels, albumin ratio, and CD4^+^ T-cell count were performed with linear mixed-effects models. Generalized estimating equations were used for analysis of CSF WBC counts of ≥ 3 × 10^6^ cells/L in relation to CSF HIV-1 RNA levels greater than the LLQ and to compare the incidence of CSFE between the present study and our prior study [12]. Correlations between biomarkers, calculated from the entire study population, were explored using the Pearson correlation coefficient. A 2-sided *P* value of < .05 was considered statistically significant. Statistics and figures were performed and made using R statistical computing software (version 4.2.3, R Core Team, 2020), SPSS (IBM SPSS version 29), or Prism 10 (GraphPad Software).

## RESULTS

### Study Population

**Figure 1.**
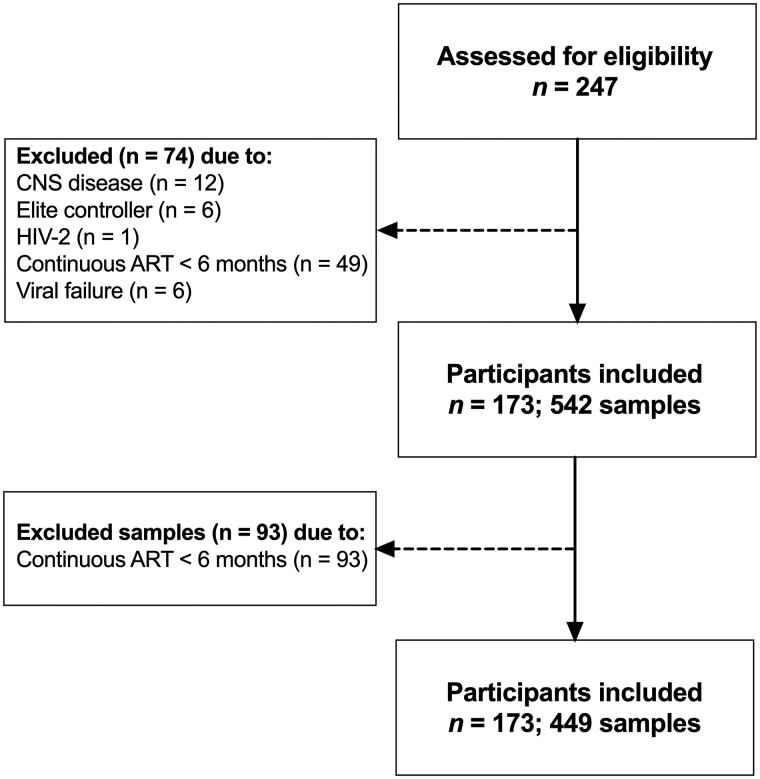
Flowchart of the study. Inclusion period spanned from April 2016 to April 2022. “Samples” refers to paired samples of blood and cerebrospinal fluid drawn on the same sampling occasion. CNS disease included participants with CNS opportunistic infections and cerebrovascular insults < 1 year prior to sampling. Abbreviations: ART, antiretroviral therapy; CNS, central nervous system; HIV-2, human immunodeficiency virus type 2.

**Figure 2.**
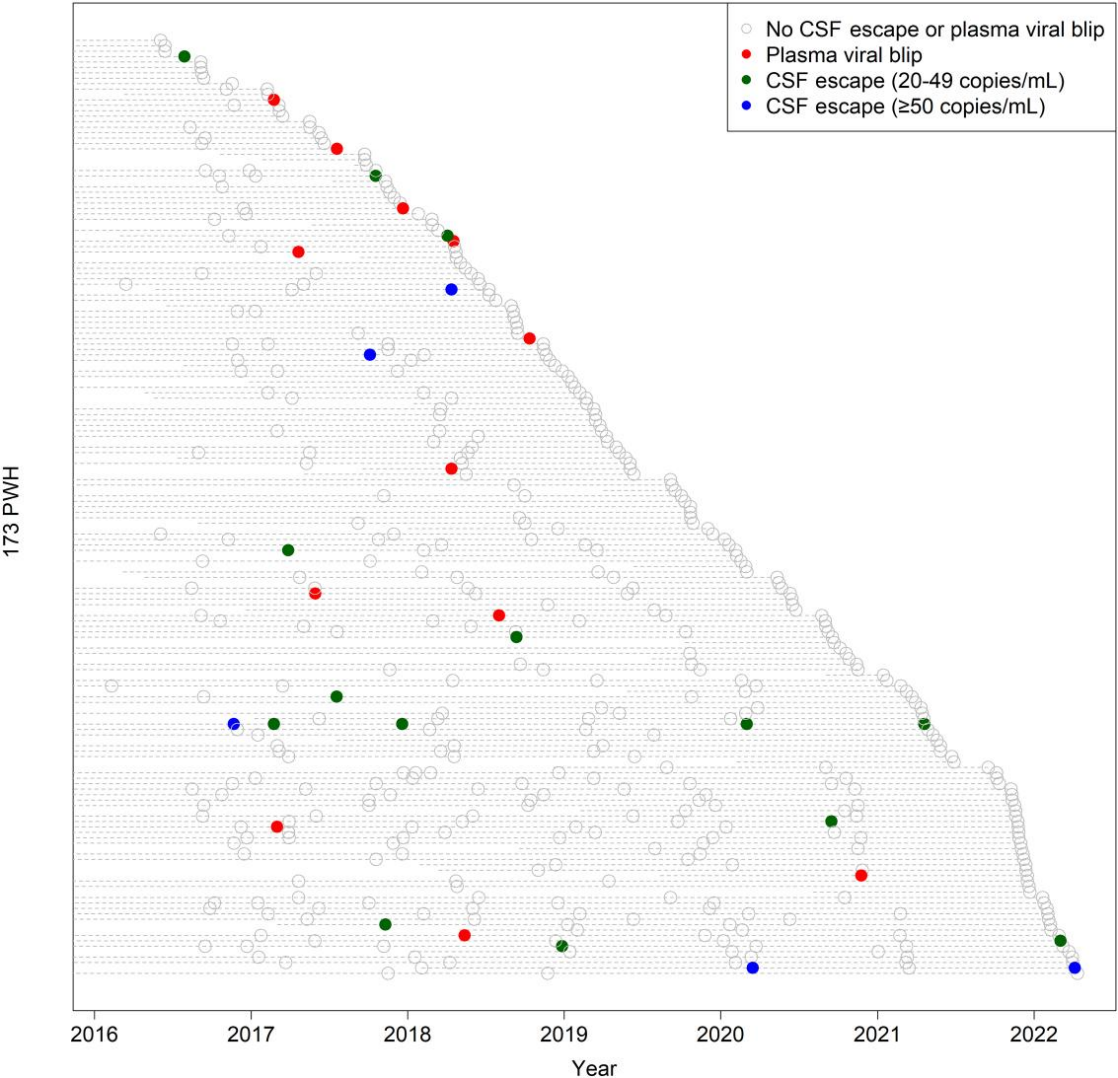
Longitudinal overview of CSF escape and plasma viral blips (blips) in the study population. The x-axis represents the study period. The 173 study participants are plotted individually on the y-axis and represented by dashed horizontal lines longitudinally from the time of entry into the study. Each circle represents an individual sample. Empty grey circles indicate HIV-1 RNA < 20 copies/mL in both plasma and cerebrospinal fluid, blue filled circles indicate asymptomatic CSF escape (cutoff 50 copies/mL for CSF HIV-1 RNA), green filled circles indicate asymptomatic CSF escape with CSF HIV-1 RNA within the quantification range of 20-49 copies/mL, and red filled circles indicate plasma viral blips (HIV-1 RNA ≥50 copies/mL). The total number of samples was 449. The mean number of samples per participant was 2.6 (standard deviation = 1.6). Abbreviations: CSF, cerebrospinal fluid; HIV, human immunodeficiency virus; PWH, people with HIV.

Two hundred and forty-seven PWH were assessed for eligibility, of whom 173 (that had performed 449 lumbar punctures) met the inclusion criteria (Figure 1) and were included in the analysis. Participants were mostly middle-aged (median age 47 years) and men (66%). The median total ART exposure was 74 months (IQR, 24–150 months) and 31 (18%) had a history of an AIDS-defining condition. The characteristics of the study population are shown in Table 1. The average number of samples per study participant was 2.6 (range, 1–7), with 114 participants having 2 or more samples collected (Figure 2). The median duration between samples was 377 days (IQR, 350­–430 days) and the median follow-up time for participants with multiple samples was 1035 days (IQR, 428–1530 days). Thirty-five out of the 173 (20%) participants were also included in our previous longitudinal study [12].

**Table 1.** Baseline Characteristics of the Study Population

Characteristic	CSF Escape, Cutoff 20 copies/mL (n = 14)	No CSF Escape (n = 159)	*P*
Women	3 (21)	57 (36)	.38
Age, y	49 (40–62)	47 (38–55)	.36
Time since HIV diagnosis, mo	72 (12–130)	104 (30–192)	.19
Past AIDS-defining condition	3 (21)	28 (18)	.72
ART duration, mo	53 (11–128)	78 (25–162)	.24
Duration of plasma HIV-1 RNA <50 copies/mL, mo	45 (10–112)	61 (16–112)	.45
Past CSF escape	1 (7)	17 (11)	1.0
Past treatment interruption or viral failure	2 (14)	29 (18)	.73
CD4^+^ T-cell count, cells/µL			
Nadir	250 (125–318)	230 (110–390)	.94
Current	585 (475–728)	620 (480–810)	.54
Pretreatment HIV-1 RNA, log_10_ copies/mL			
Plasma	5.3 (4.4–5.7)	4.8 (4.2–5.4)	.12
CSF	4.1 (3.5–4.7)	3.8 (3.1–4.5)	.34

Data are number (%) of participants or median value (interquartile range). Significance test used Fisher exact test (dichotomous variables) or Mann-Whitney *U* test (continuous variables).

Abbreviations: ART, antiretroviral therapy; CSF, cerebrospinal fluid.

### Incidence of CSF Escape

CSFE (cutoff 50 copies/mL) was present in 4 of 173 PWH (2%). Of the 449 samples, CSFE was detected in 5 (1%), with a median CSF HIV-1 RNA of 75 copies/mL (range, 55–924). The corresponding proportions when applying 20 copies/mL as the cutoff for CSF HIV-1 RNA, were 14 of 173 (8%) of PWH and 19 of 449 (4%) samples. One participant had an isolated increase in CSF HIV-1 RNA (≥20 copies/mL) in 5 consecutive samples (range, 20–55 copies/mL) but remained neuroasymptomatic and clinically stable throughout the study period. Similarly, all the PWH with CSFE remained neuroasymptomatic.

**Figure 3.**
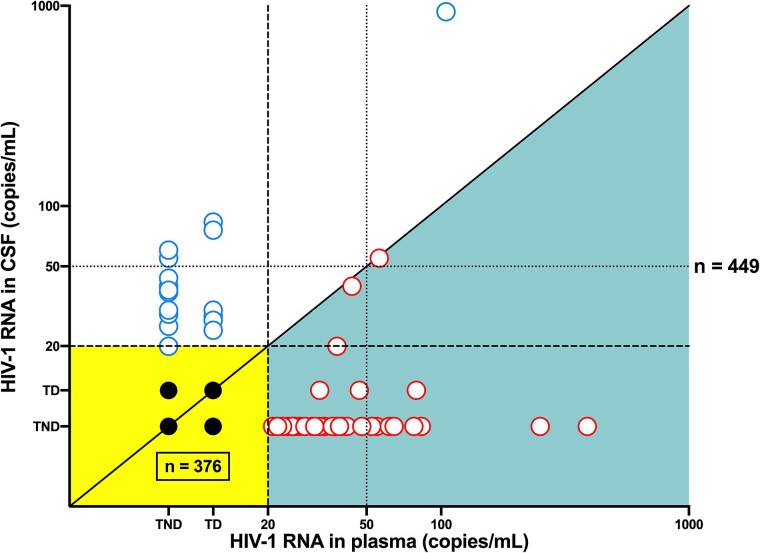
Relationship between plasma HIV-1 RNA (x-axis) and CSF HIV-1 RNA (y-axis) among the 449 samples. The densely dashed line indicates the LLQ of 20 copies/mL and the dotted line indicates the widely utilized cutoff of 50 copies/mL. The 4 black filled circles (yellow background) represent the 376 samples where HIV-1 RNA levels were below LLQ in both compartments. The empty blue circles (white background) represent samples with CSF HIV-1 RNA > LLQ and greater than plasma HIV-1 RNA (n = 19). The empty red circles (turquoise background) represent samples with plasma HIV-1 RNA > LLQ and greater than CSF HIV-1 RNA (n = 54). TD in both compartments, n = 4; TD in plasma and TND in CSF, n = 86; TD in CSF and TND in plasma, n = 3; TND in both compartments, n = 283. Abbreviations: CSF, cerebrospinal fluid; LLQ, lower limit of quantification; TD, target detected but not quantifiable; TND, target not detected.

For comparison, 12 participants had 1 or more plasma blips when the cutoff of 50 copies/mL was applied (7%), and 46 participants when the cutoff of 20 copies/mL was used (26%). Of the 449 samples, plasma viral blips occurred in 12 (cutoff 50 copies/mL; 3%) or 54 samples (cutoff 20 copies/mL; 12%) (Figure 3). None of the 4 PWH with CSFE had any plasma viral blip (≥50 copies/mL). CSFE with concurrent quantifiable HIV-1 RNA in plasma was only found in 1 of 5 samples (CSF HIV-1 RNA of 924 copies/mL and plasma HIV-1 RNA of 104 copies/mL).

We also compared the incidence of CSFE in this study to the historical cohort from 1997 to 2015 [12], applying the CSFE definition used in the present study (cutoff 50 copies/mL). Of note, CSFE was identified in 16 of 75 PWH (21%) and in 19 of 418 (5%) samples in the historical cohort, compared to 4 of 173 PWH (2%) and 5 of 449 samples (1%) in the present study. Using generalized estimating equations accounting for individuals that were tested repeatedly and/or participated in both studies (n = 35), we observed a significantly reduced occurrence of CSFE in the samples of the present study (odds ratio = .24, *P* = .01).

### Longitudinal Analyses of CSF and Blood Biomarkers

The median averaged CSF neopterin level in the study population was 6.2 mmol/L (IQR, 5.0–7.8). In a linear mixed-effects model analysis of the log_10_ CSF neopterin level, samples demonstrating CSFE (cutoff 20 copies/mL) had a 25% higher geometric mean CSF neopterin compared to samples without (*P* = .01). No differences in plasma neopterin were found between samples with and without CSFE (cutoff 20 copies/mL; *P* = .9). Similarly, no significant differences in plasma neopterin were observed between samples with or without plasma viral blips.

The median averaged albumin ratio in the study population was 4.7 (IQR, 3.4–5.6). In a linear mixed-effects model analysis of log_10_ albumin ratio, samples with CSFE (cutoff 20 copies/mL) had an 8% higher geometric mean albumin ratio compared to samples without (*P* = .04). Albumin ratio was above the upper normal reference value in only 1 of the 19 samples with CSFE (cutoff 20 copies/mL).

The median averaged age-adjusted CSF NfL levels in the study population was 445 ng/L (IQR, 351–628 ng/L). In a linear mixed-effects model analysis of log_10_ age-adjusted CSF NfL, no significant difference was identified in relation to the presence or absence of CSFE (cutoff 20 copies/mL; *P* = .8). However, a weakly significant correlation was seen between CSF neopterin and CSF NfL (*r* = .12, *P* = .01) as well as between CSF neopterin and albumin ratio (*r* = .17, *P =* .001) in the entire study population.

No significant differences were seen in CD4^+^ T-lymphocyte count with respect to the presence or absence of CSFE (cutoff 20 copies/mL; *P* = .43; Table 1). Using a generalized estimating equations analysis, the odds ratio for having a detectable CSF WBC count (≥ 3 × 10^6^ cells/L) when CSFE (cutoff 20 copies/mL) was present was 3.9 (*P* = .004).

### Possible Predictors of CSF Escape

Due to the low incidence of CSFE (cutoff 50 copies/mL), it was not possible to perform statistical comparisons of baseline characteristics between the groups with and without CSFE. When comparing the groups with and without CSFE applying 20 copies/mL as the cutoff for CSF HIV-1 RNA, no statistically significant differences were observed in baseline characteristics (Table 1). As expected, the frequency of per-patient samplings was notably higher among PWH with CSFE in comparison with those without CSFE (*P* = .01, mean 3.6 [SD, 1.5] and 2.5 [SD, 1.5], respectively). The distribution of ART regimens in the overall study population as well as during CSFE (cutoff 20 copies/mL) is shown in Table 2. The composition of ART drugs varied over time where 46 out of 114 longitudinally sampled PWH (40%) changed ART regimen during the study period. The variation in ART regimens did not allow for a conclusive analysis of the impact of individual drugs on CSF HIV-1 RNA or biomarkers. Nearly half (47%) of the cohort had an integrase strand transfer inhibitor (INSTI)-containing regimen on at least at 1 sample occasion.

**Table 2.** Overview of ART Regimens in the Study Population

ART Regimen	Total (n = 449)	CSF Escape, Cutoff 20 copies/mL (n = 19)
PI + ritonavir/cobicistat + NRTIs	104 (23)	4 (21)
NNRTI + NRTIs	87 (20)	9 (47)
INSTI + NRTIs	212 (47)	6 (32)
Other^a^	46 (10)	…

Data are number (%).

Abbreviations: ART, antiretroviral therapy; INSTI, integrase strand transfer inhibitor; NNRTI, nonnucleoside reverse transcriptase inhibitor; NRTI, nucleoside reverse transcriptase inhibitor; PI, protease inhibitor.

^a^Including INSTI + NNRTI, PI + INSTI + NNRTI + NRTIs, PI + INSTI + NRTIs, PI + INSTI ± maraviroc, and triple NRTI.

## DISCUSSION

In the present analysis we conducted a longitudinal study of a large, well-characterized PWH cohort using contemporary ART regimens to gain a better understanding of the occurrence and nature of asymptomatic CSFE [15, 28, 29]. Compared to our previous longitudinal study, asymptomatic CSFE was less common (2% vs 21%), with only 1 participant exhibiting repeatedly quantifiable CSF HIV-1 RNA measurements. Among these, only 1 of 5 samples had CSF HIV-1 RNA levels ≥50 copies/mL. In accordance with prior studies, an association was identified between quantifiable CSF HIV-1 RNA and biomarkers of inflammation (CSF neopterin and CSF WBC), but not with the neuronal injury marker CSF NfL [11, 12, 16–18]. Additionally, we also observed an association between quantifiable CSF HIV-1 RNA and blood-brain barrier (BBB) integrity (albumin ratio). Overall, these associations support our previous studies indicating that the presence of low levels of HIV-1 RNA in the CSF compartment still elicits an intrathecal immune response despite clinically effective ART.

Advances in HIV therapy, encompassing both earlier ART initiation (with correspondingly higher CD4 nadir), less frequency and shorter duration of suboptimal ART, and the more widespread use of INSTIs, may explain the reduced incidence of asymptomatic CSFE observed in the present study compared to our previous longitudinal study. Furthermore, the lower incidence of plasma blips might reflect better adherence, possibly due to less complex regimens and less ART side effects [33]. In our previous longitudinal study, only 1 of 16 participants had CSFE (cutoff 50 copies/mL) in repeated measurements. We therefore proposed that asymptomatic CSFE, in most cases, reflected low-level variations in virus release, analogous to plasma blips, rather than active CNS infection [12]. The findings of the present study support this hypothesis, where none had CSFE (cutoff 50 copies/mL) in repeated measurements.

The current study confirmed the association previously described between the occurrence of quantifiable CSF HIV-1 RNA and elevated CSF neopterin levels. Neopterin is a member of the pteridine family and is mainly released from macrophages, microglia, and monocytes in response to interferon-γ [30]. Its concentration in CSF is closely correlated to CSF viral load in untreated HIV but it has also been shown to correlate well with low-level viremia during suppressive ART and is therefore considered a valuable biomarker for immune activation even in the ART era [30, 34–39]. In untreated infection, CSF neopterin has also been shown to correlate with CSF NfL, a sensitive biomarker of axonal injury [32]. This has raised concerns that the low-grade CSF immune activation often observed even in virally suppressed PWH may lead to neuronal damage [40, 41]. Of note, we did not observe any significant association between quantifiable CSF HIV-1 RNA and elevated levels of CSF NfL, which was in accordance with prior studies [12, 18]. However, the correlation found between CSF neopterin, and CSF NfL suggests that inflammation-driven neuronal damage cannot be entirely ruled out even with virally suppressive ART. Although the participants included in the current analysis were followed longitudinally in median 1035 days, it remains a possibility that the follow-up time was too short to detect a potential association between the presence of HIV-1 RNA and subsequent neuronal injury. Additionally, other non-HIV aspects such as lifestyle factors may contribute to the abovementioned biomarker changes [42, 43].

We also observed a higher incidence of elevated CSF WBC in samples with quantifiable CSF HIV-1 RNA. CSF pleocytosis, primarily comprising lymphocytes, has previously been demonstrated as a predictor of CSFE [11, 27, 44, 45]. Additionally, we observed a weak association between the occurrence of quantifiable CSF HIV-1 RNA and higher albumin ratio, although all but 1 measurement was within the normal range. Results have been conflicting regarding an association between CSFE and BBB integrity (as measured by albumin ratio or total CSF protein), and the potential impact of CSFE on BBB integrity remains to be determined [11, 18, 27, 45]. However, previous studies have consistently demonstrated an association between the albumin ratio and CSF NfL also in treated HIV, thereby highlighting that the importance of BBB function as a critical factor in determining whether low-grade viral presence can lead to neuronal impact or injury [46, 47].

We could not identify any significant predictors for asymptomatic CSFE (cutoff 20 copies/mL) among the included baseline characteristics, although this could potentially be a consequence of the small number of CSFE relative to the overall sample size. ART duration, duration of HIV infection, prior ART interruptions or failure, CD4^+^ nadir and current CD4^+^ lymphocyte count have previously been associated with CSFE to varying degrees [11, 17, 18, 27, 45]. Of note, the 14 PWH with CSFE (cutoff 20 copies/mL) had a higher number per-patient samples compared to the remaining 159 participants, which probably increased the likelihood of detecting instances of isolated CSFE.

The major strength of this study is its large-scale longitudinal analysis of asymptomatic CSFE, a design often highlighted as necessary in previous cross-sectional studies [11, 18]. Additionally, all samples were taken as part of a research protocol, in contrast to other studies where sampling was performed on clinical indications related to neurological symptoms. However, we acknowledge several limitations. The median sampling interval of 377 days prohibited closer evaluation of the duration of CSFE. Furthermore, as mentioned previously, it is possible that the varying sampling intervals of the participants influenced the ability to detect associations between CSFE and later biomarker or clinical alterations. Additionally, we cannot exclude a selection bias in our cohort, which may limit the generalizability of the results and potentially influence the observed incidence of CSFE in the broader HIV population.

With 1 notable exception, CSF HIV-1 RNA levels were low and often approached the LLQ. Consequently, Poisson variations might have influenced the PCR results. We aimed to reduce bias due to interassay variation by implementing the same HIV-1 RNA assay throughout the study but inherent intraassay variation is a well-known issue that may have impacted the results [48, 49]. However, the association between quantifiable CSF HIV-1 RNA and CSF neopterin, but not plasma neopterin, observed in this and previous studies strongly suggests that the phenomenon is not solely due to intraassay variability.

Our study indicates that asymptomatic CSFE is rare with current ART, and that contemporary guidelines promoting prompt initiation of ART upon diagnosis, possibly coupled with more potent antiretroviral drugs, have contributed to a lower incidence of asymptomatic CSFE than previously reported. Asymptomatic CSFE appears clinically benign, and our results do not support any modifications of otherwise effective ART regimens in cases with asymptomatic CSFE. However, increased watchfulness for newly emerging neurological symptoms might be prudent. Considering the nature of the CNS as a viral reservoir, further exploration of genetic populations in the CSF remains of great importance.

In conclusion, in this longitudinal study of neuroasymptomatic PWH on effective ART, we found that asymptomatic CSFE was less frequent than we have previously reported, typically with low CSF viral load and none of the participants had consecutive samples with CSFE. Hence, we argue that our earlier suggestion [12] to classify these temporary isolated elevations in CSF HIV-1 RNA as CSF viral blips, rather than CSF escape, is justified and the term asymptomatic CSF escape should only be used in situations involving repeated isolated elevations of CSF HIV-1 RNA.

## Notes

***Author contributions.*** M. G. and A. E. originated the idea and supervised the study. G. U., M. G., and A. E. were responsible for the study design, contributed to the acquirement, analysis, and interpretation of data, and wrote the article. M. G., A. E., A. Y., Å. M., E. T., E. S., and L. H. recruited the participants. J. G., D. F., H. Z., and K. N. performed biochemical analyses. G. U., S. N., A. E., and M. G. did the statistics. M. G. was responsible for securing the funding for the study. All authors contributed to the manuscript preparation.

***Disclaimer.*** The funding body did not influence the study design, data collection, data analysis, interpretation of data, or the writing process.

***Financial support.*** This work was supported by grants from the Swedish state under the agreement between the Swedish government and the county councils, the ALF-agreement (grant numbers ALFGBG-965885 and ALFGBG-71320). H. Z. is a Wallenberg Scholar and a Distinguished Professor at the Swedish Research Council.
